# Determination of Pharmacokinetic and Pharmacokinetic-Pharmacodynamic Parameters of Doxycycline against *Edwardsiella ictaluri* in Yellow Catfish (*Pelteobagrus fulvidraco*)

**DOI:** 10.3390/antibiotics10030329

**Published:** 2021-03-21

**Authors:** Ning Xu, Miao Li, Xiaohui Ai, Zhoumeng Lin

**Affiliations:** 1 Yangtze River Fisheries Research Institute, Chinese Academy of Fishery Sciences, Wuhan 430223, China; xuning@yfi.ac.cn; 2 Institute of Computational Comparative Medicine (ICCM), Department of Anatomy and Physiology, College of Veterinary Medicine, Kansas State University, Manhattan, KS 66506, USA; miaoli@ksu.edu; 3 Hu Bei Province Engineering and Technology Research Center of Aquatic Product Quality and Safety, Wuhan 430223, China; 4 Key Laboratory of Control of Quality and Safety for Aquatic Products, Ministry of Agriculture and Rural Affairs, Beijing 100141, China

**Keywords:** doxycycline, pharmacokinetics, pharmacokinetic–pharmacodynamic (PK/PD) parameters, *Edwardsiella ictaluri*, yellow catfish

## Abstract

This study aimed to examine the pharmacokinetics of doxycycline (DC) in yellow catfish (*Pelteobagrus fulvidraco*) and to calculate related pharmacokinetic–pharmacodynamic (PK/PD) parameters of DC against *Edwardsiella ictaluri*. The minimum inhibitory concentration of DC against *E. ictaluri* was determined to be 500 µg/L. As the increase of oral dose from 10 to 40 mg/kg, the area under the concentration vs. time curve from 0 to 96 h (AUC_0–96_) values were considerably increased in gill, kidney, muscle and skin, and plasma, except in liver. C_max_ values exhibited a similar dose-dependent increase trend in plasma and tissues except in liver, but other PK parameters had no apparent dose-dependence. The PK/PD parameter of the ratio of AUC_0–96_ to minimum inhibitory concentration (AUC_0–96h_/MIC) was markedly increased in plasma and tissues dose-dependently except in liver, but %T > MIC values were increased only moderately at some dose groups. After receiving the same dose with disparate time intervals from 96 to 12 h, the AUC_0–96h_/MIC was distinctly increased in plasma and tissues, but the %T > MIC had a decreasing trend. When administering 20 mg/kg with a time interval of 96 h, the AUC_0–96h_/MIC values were consistently >173.03 h and the %T > MIC values were above 99.47% in plasma and all tissues. These results suggest that administration of DC at 20 mg/kg every 96 h is a preferable regimen in yellow catfish.

## 1. Introduction

Yellow catfish (*Pelteobagrus fulvidraco*) is a predominant cultured fish species in China with a total production of more than 0.48 million tons per year [1]. To pursue a high yield, the culture density of yellow catfish per 1 m^3^ is increased continuously, which makes it easy to cause an outbreak of bacterial diseases, especially *Edwardsiella ictaluri* infection. Clinical manifestations of *Edwardsiellosis* are mainly classified as an acute type and a chronic type [2,3]. The acute type has a higher mortality that is infected from the digestive tract to blood and various organs to cause organ hyperemia, hemorrhage, inflammation, denaturation, necrosis, and ulceration. The typical symptom is a sick fish hanging in the water with head up, tail down, sometimes in spasmodically spiral swimming, and leading to death. The chronic type has a longer course than the acute type. The pathogen invades the olfactory bulb through the nasal cavity, then travels to the brain, and finally reaches the skull through the meninges and the skin of head. The typical symptoms are skin necrosis and ulceration and the formation of an open ulcer on the head, known as “head hole disease” [3]. Due to the widespread infection in the fish body of *E. ictaluri*, an aquatic drug with a high permeability is needed to cure the disease. In clinical therapy, the first selected drug is sulfadiazine, which can penetrate the blood–brain barrier to reach the brain, but its therapeutic efficacy is rapidly decreasing because of serious drug resistance [4]. Fortunately, it has been found that doxycycline (DC) is an ideal choice among the approved drugs due to its good penetration properties in the tissues [5,6,7].

Doxycycline (DC), a member of second-generation tetracyclines, has been extensively used in global aquaculture due to better chemical properties of plasma half-lives, lipid solubility, and antibiotic activity than its analogs [8,9]. DC is also approved in aquaculture against *Aeromonas hydrophila*, *E. ictaluri*, *Fibrobacter columnaris*, *Pseudomonas fluorescens*, and *Vibrio vulnificus* in China [2,10,11,12,13]. Currently, multiple pharmacokinetic (PK) and residue depletion studies of DC are available in tilapia [14] and grass carp [15,16]. These studies reported that DC had a plasma elimination half-life of >20 h in grass carp following a single oral dose at 20 mg/kg, and of 39 h in tilapia following a single intravenous dose at 20 mg/kg; these relatively long plasma half-lives were in part caused by enterohepatic recycling [14,15,16]. For the purpose of fish health, it is important to establish an efficient therapeutic regimen for specific fish species based on pharmacokinetic–pharmacodynamic (PK/PD) studies. Some PK/PD studies have been performed in veterinary animals for optimizing DC’s therapeutic regimen. For example, a PK/PD study of DC was carried out in *Mycoplasma gallisepticum*, which causes chronic respiratory disease in chickens using an in vitro dynamic model [17]. The estimated %T > MIC values for 0log10 (CFU/mL), 2log10 (CFU/mL) reduction, and 3log10 (CFU/mL) reduction were 32.48%, 45.68%, and 54.36%, respectively. This study showed good effectiveness and time-dependent characteristics of DC against *M. gallisepticum* in vitro [17]. Zhang and colleagues reported DC’s optimum dosage regime against *Haemophilus parasuis* in pigs based on PK/PD integration modeling [18]. According to values of AUC_0–24 h_/MIC, the doses predicted to obtain bacteriostatic, bactericidal, and elimination effects for *H. parasuis* over 24 h were 5.25, 8.55, and 10.37 mg/kg for the 50% target attainment rate (TAR), and 7.26, 13.82, and 18.17 mg/kg for 90% TAR, respectively [18]. However, there are no PK/PD studies reported in any specific fish species. Furthermore, limited PK/PD information on DC concerning *E. ictaluri* is available in yellow catfish.

The objective of this study was to investigate the pharmacokinetics of DC in yellow catfish at different oral doses and to calculate related PK/PD parameters of DC against *E. ictaluri*. The results will provide useful information to optimize the dosing regimen of DC against *E. ictaluri* in yellow catfish.

## 2. Results

### 2.1. In Vitro Susceptibility Assay

The average MIC of DC against *E. ictaluri* was 500 µg/L in yellow catfish plasma.

### 2.2. Analytical Method Validation

The limit of detection and the limit of quantification of DC were determined to be 25.0 and 50.0 μg/L (or μg/kg), respectively, in plasma and tissues. The matrix-match calibration curves were established across spiked concentrations from 50 to 2000 μg/L or μg/kg in plasma and tissues, and a good linearity was achieved with the coefficient of correlation R^2^ = 0.999. If DC’s concentrations in some samples were found to be more than the upper limit of quantification, the remaining samples were repeatedly determined after diluting with the corresponding blank samples. The results of mean recovery rates for DC ranged from 67.2% to 83.7% in plasma and tissues (Table 1). The percentages of relative standard deviations for inter-day and intra-day precision were ≤10% (Table 1).

### 2.3. PK Profile of DC in Yellow Catfish

**Figure 1:**
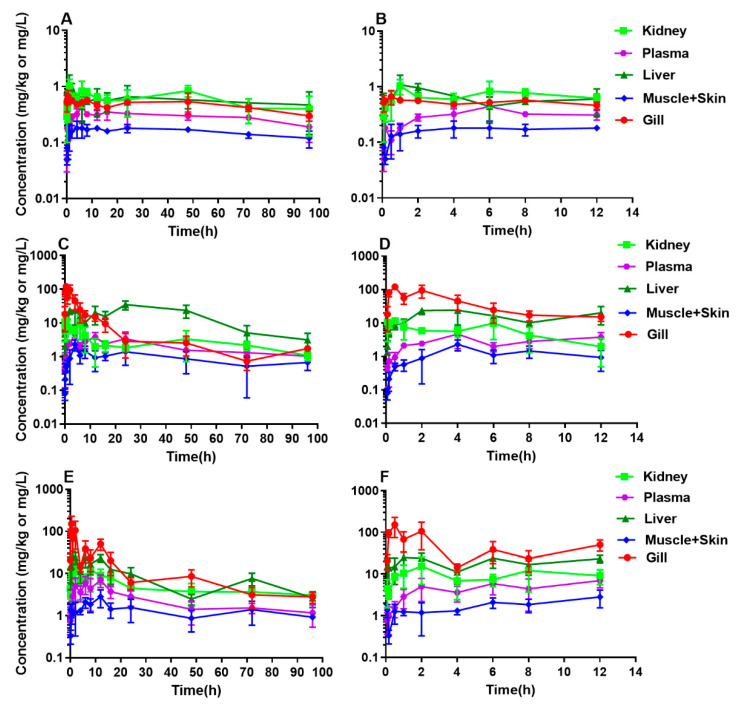
Semi-logarithmic plots of plasma and tissue concentration-time profiles of doxycycline in yellow catfish (*Pelteobagrus fulvidraco*) following an oral dose at 10 (**A**,**B**), 20 (**C**,**D**), or 40 mg/kg (**E**,**F**) at 24 °C. **A**, **C**, and **E**: For all sampling time points; **B**, **D**, and **F**: For a part of the sampling points from 0.083 to 12 h. Sample size: *n* = 6.

The DC concentration vs. time profiles in plasma and tissues of yellow catfish after a single oral administration at different doses of 10, 20, and 40 mg/kg are shown in Figure 1. All raw concentration data of DC are provided in supplementary Tables S1–S3. Generally, DC concentrations in plasma and tissues were increased along with the rise of the given dose level from 10 to 40 mg/kg, especially in gill. An interesting finding was a multiple-peak phenomenon in the concentration-time curves of plasma and tissues. From 0.5 to 48 h post-dosing of 10 mg/kg, the concentrations of DC in plasma and tissues fluctuated, and then gradually decayed (Figure 1 and Table S1). At 20 mg/kg, the levels in plasma and tissues also fluctuated from 0.5 to 48 h and then displayed a decreased trend (Figure 1 and Table S2). After a given dose of 40 mg/kg, the time period of the concentration undulation was further enlarged from 0.08 to 72 h (Figure 1 and Table S3). Overall, the time range of concentration fluctuation at lower doses in various tissues was shorter than that at the highest dose.

Table 2 shows all calculated PK parameters. With the increase of the given dose from 10 to 40 mg/kg, the C_max_ values were increased from 0.66 to 151.94 mg/kg in gill, from 1.03 to 15.40 mg/kg in kidney, from 0.18 to 2.84 mg/kg in muscle and skin, and from 0.44 to 6.99 mg/L in plasma, except in liver, which was firstly increased from 1.08 to 34.81 mg/kg, then decreased to 24.95 mg/kg. The AUC_0-96_ values exhibited similar increasing trends in gill, kidney, muscle and skin, and plasma, but not in liver. The values of λ_z_, T_1/2 λz_, T_max_, V_z__F, and CL_F did not present apparent dose-dependence. The AUC_%extrap values were higher than 20% in plasma and muscle and skin in all dose groups and in all tissues in the 10 mg/kg dose group.

### 2.4. PK/PD Integration for DC in Plasma and Tissues

Table 3 presents the PK/PD parameters of AUC_0–96h_/MIC and %T > MIC by the integration of PK data and the MIC value using a non-parametric superposition approach. As the increase of the oral dose level from 10 to 40 mg/kg, the values of AUC_0–96h_/MIC were notably increased in plasma and tissues except for liver. The values of %T > MIC were markedly increased along with the dose increase from 10 to 20 mg/kg. Once the dose was over the threshold of 20 mg/kg, they remained constant in plasma and each tissue. At the scenario of an identical oral dose with changing the administration interval from 96 to 12 h, the values of AUC_0–96h_/MIC were considerably increased. For example, at a dose of 10 mg/kg, its values raised from 55.72 to 265.29 h in plasma, 89.37 to 425.79 h in gill, from 114.47 to 555.15 h in kidney, from 109.30 to 516.17 h in liver, and 30.43 to 143.61 h in muscle and skin. However, the values of %T > MIC had a declining trend. Furthermore, the values of %T > MIC were below 78.0% at an oral dose of 10 mg/kg. When the administration dose was increased to 20 mg/kg, %T > MIC ranged from 99.5% to 100.0% in plasma and tissues at an administration interval of 96 h, but showed no improvement along with the decrease of dose intervals.

## 3. Discussion

*E. ictaluri* is an important pathogen in global aquaculture, particularly in the culture of yellow catfish and channel catfish, and it causes a great economical loss every year. However, the therapeutic information of the concerned drug based on PK/PD indices is scarce. In this study, we evaluated the PK/PD parameters of a candidate drug of DC against *E. ictaluri* in yellow catfish based on the MIC value and PK parameters following different single oral doses at 10, 20, and 40 mg/kg, respectively. This study provides useful information for the effective use of DC in yellow catfish against *E. ictaluri.*

To obtain sufficient pharmacological information on DC, PK studies of DC were performed in yellow catfish at different single oral doses. According to observed results, an obvious multiple-peak phenomenon was found in DC concentration vs. time curves in plasma and tissues, which was consistent with the results in grass carp (*Ctenopharyngodon idella*) and tilapia (*Oreochromis aureus × Oreochromis niloticus*) following a single oral dose at 20 mg/kg at 24 °C [14,16]. In addition, DC displayed multiple peaks in PK profiles in ducks [19], pigs [20], and humans [21]. This multiple-peak feature could be partly due to the impact of enterohepatic recycling because DC might form stable complexes with bile and enter the intestine via the biliary excretion to be reabsorbed into liver after digestion [8]. 

The PK parameter of T_1/2λz_ ranged from 16.27 to 56.55 h in gill, from 29.60 to 143.26 h in kidney, from 16.49 to 142.52 h in liver, from 82.31 to 147.18 h, and from 80.81 to 106.38 h in plasma after a single oral dose at different levels (10, 20, or 40 mg/kg). These data did not present an apparent dose-dependence of T_1/2λz_ with the increased dose of DC but showed a large difference in the same tissue among different given dose levels. These discrepancies may be possibly due to the calculation method used in the software Phoenix. The T_1/2λz_ was calculated using the equation of T_1/2λz_ = 0.693/λ_z_. The value of λ_z_ is a linear slope of the kinetic profile at the terminal elimination phase. In Phoenix, there are two calculation approaches for λ_z_, one is the best slope identified automatically by the Phoenix software, and another is to manually choose three or more time points to perform the calculation [16]. In the present study, the authors chose the former method to calculate λ_z_ without manual adjustments. In addition, due to the multiple-peak phenomenon, the selected time points for calculation in each tissue were different, which, in part, caused the differences in values of λ_z_. Consequently, T_1/2λz_ in the same tissue under disparate doses presented different values. 

In addition to the increase of oral dose, the C_max_ also presented an increasing trend in all tissues except in liver. The exact reason for the lack of a dose-dependent increase in the Cmax of liver is unknown. Only the value of C_max_ in gill was higher than grass carp, but the values in other tissues and plasma were all smaller than grass carp by oral administration at the same dose at 24 °C [16]. The calculated T_max_ values ranged from 0.5 to 24 h following different single DC doses of 10, 20, and 40 mg/kg, which did not exhibit apparent regularities in each tissue as the rise of the dose. At the dose of 20 mg/kg, T_max_ values ranged from 0.5 to 24 h in plasma and tissues except for gill. These values were longer than the corresponding values in grass carp receiving the same dose at 24 °C [16]. Moreover, there was also no obvious dose-dependence in V_z__F values and CL_F values accompanying the increase of administration dose. The V_z__F value (7.47 L/kg) at the dose of 20 mg/kg in yellow catfish was notably higher than that in tilapia (2.32 L/kg) [14] and grass carp (0.87 L/kg) [16] following the same oral dose at identical water temperature, suggesting that the distribution of DC in yellow catfish was more widely than tilapia and grass carp. The CL_F value in yellow catfish (0.06 L/h/kg) was larger than the corresponding values in tilapia (0.04 L/h/kg) [14] and grass carp (0.03 L/h/kg) [16]. Finally, the values of AUC_0-96_ exhibited an increasing trend with the rise of given dose in gill, kidney, muscle and skin, and plasma, but its value was firstly increased (at a dose from 10 to 20 mg/kg) and then decreased (at a dose from 20 to 40 mg/kg) in liver. The exact reasons for this phenomenon are not known. The AUC_%extrap values were consistently higher than 20% in the plasma and muscle and skin for all dose groups and in all tissues in the 10 mg/kg dose group. This is a limitation of this study and these results suggest that the sensitivity of the analytical method was not good enough and/or the sampling duration was not long enough; thus, the calculation of the half-life values could be inaccurate. Future studies using more sensitive detection methods with longer sampling duration are needed to more accurately calculate the half-life of DC in yellow catfish.

For the purpose of reducing the number of experimental animals, this study used a non-parametric superposition approach with the Phoenix software to simulate the PK profiles after multiple oral doses with different time intervals based on the PK parameters from a single oral dose [22]. Phoenix’s non-parametric superposition object is based on non-compartmental results describing single-dose data to predict drug concentrations after multiple doses at a steady state. The predictions are on the basis of an accumulation ratio calculated from the terminal slope, which can be used for simple (the same dose was given in a constant interval) or complicated dosing schedules (based on Phoenix WinNonlin User’s Guide). The simulated results can help design optimal dosage regimes or predict outcomes of clinical trials when used in conjunction with the semi-compartmental modeling function. In actual PK studies, the non-parametric superposition approach has been extensively used [23,24,25]. Its assumptions are typically as follows: (a) Application of linear PK to accommodate a change in dose during the multiple dosing regimen; (b) each dose of a drug acts independently of every other dose; (c) the rate of absorption and the average systemic clearance are consistent for each dosing interval [25].

Regarding the pharmacodynamic component of this study, the parameter of MIC for DC against *E. ictaluri* was measured in yellow catfish plasma. It has been reported that the MIC value measured in the broth was conspicuously different from that measured in plasma [26,27]. A study found that the MIC value of enrofloxacin in plasma countering *A. hydrophila* was remarkably higher than that in broth [28]. The authors proposed that, if different MICs were found in broth and plasma, the corresponding adjustment should be performed by a scaling factor when the PK/PD breakpoint indices were used to optimize dosages. Furthermore, the in vitro susceptibility of macrolides and ketolides also manifested a marked enhancement of antibiotic activity against *Pseudomonas aeruginosa* in RPMI 1640 medium [29]. Therefore, the matrix between broth and plasma may influence antimicrobial activity, and it is better to use plasma for dilution and incubation of bacteria to determine the MIC because the composition of plasma is the closest to the in vivo environment. 

DC possesses a high lipophilicity and permeability that can result in high concentrations in various tissues after oral administration [8]. This feature is beneficial for treating infectious diseases. Generally, DC is considered a time-dependent drug. A previous study showed that DC presented time-dependent killing for *M. gallisepticum* in an in vitro model [17]. Cunha and co-workers also reported that DC exhibited a time-dependent killing at low concentrations of 2–4 times the MIC, but a concentration-dependent killing at high concentrations of 8–16 times the MIC against *Staphylococcus aureus*, *Streptococcus pneumoniae*, *Escherichia coli,* and *Pasteurella multocida* [30]. However, a PK/PD study of DC against *H. parasuis* directly showed a dose-dependent property [18]. These discrepancies may be caused by different target pathogens. This viewpoint has been proven in the PK/PD study of gentamicin, which displayed a time-dependent kinetic profile for countering *S. aureus,* but a concentration-dependent kinetic profile against *Pseudomonas aeruginosa* [31]. In this study, one limitation was that the in vitro killing curve was not determined. As a result, we were unable to establish the PK/PD correlation based on the sigmoid inhibitory *Emax* model. Nevertheless, the present study provides valuable information on AUC/MIC and %T > MIC using the non-parametric superstition approach based on PK characteristics at different single oral doses. 

AUC/MIC and %T > MIC are important PK/PD indices for establishing or optimizing the dosage regimen. In this study, with the increase of the given dose from 10 to 40 mg/kg, AUC/MIC values were considerably increased in plasma and each tissue except for liver. When the given dose was increased from 10 to 20 mg/kg, %T > MIC values were notably increased in plasma and tissues (e.g., gill, increased from 43.99% to 100.0%). However, when the dose was increased from 20 to 40 mg/kg, no obvious changes for %T > MIC occurred in plasma and tissues (e.g., gill, from 100.0% to 100.0%). From these results, the AUC/MIC have a concentration-dependent effect along with the increase of DC dose in plasma and tissues except in liver, but the %T > MIC was only increased moderately at certain dose levels. If the dosage was over a threshold (e.g., 20 mg/kg), it would remain at a constant. Previous studies have demonstrated that the AUC/MIC ratio of 100–125 is recommended to achieve a higher therapeutic efficacy [32,33,34]. In this study, the ratios of AUC_0–96_/MIC were more than 173.03 in plasma and tissues after oral administration at a dose of 20 mg/kg with the time interval of 96 h. In addition, with the increase of frequency of the given dose from every 96 h to every 12 h, AUC_0–96_/MIC values were increased by 409.7%–563.7% in plasma and tissues. At the given dose of 20 mg/kg with a time interval of 96 h, %T > MIC values were greater than 99.0%. However, along with the increase of administration frequency, %T > MIC values exhibited a declined tendency. These results indicate that the antimicrobial activity of DC is not necessarily proportional to the frequency of administration. Therefore, we speculate that DC presents time-dependence and %T > MIC is a more suitable PK/PD index for DC against *E. ictaluri* in yellow catfish. 

## 4. Materials and Methods

### 4.1. Chemicals and Reagents

The doxycycline (DC) standard (purity ≥ 98%) for instrument analysis was purchased from Dr. Ehrenstorfer GmbH. (Augsburg, Germany). The DC powder (purity ≥ 98%) used for oral gavage was purchased from Zhongbo Aquaculture Biotechnology Co. Ltd. (Wuhan, China). The liquid reagents of water, acetonitrile, and formic acid were obtained from Thermo Fisher (Waltham, USA) and J–T Baker (Philipsburg, USA). Ethylenediaminetetraacetic acid disodium (EDTA-Na_2_), sodium dihydrogen phosphate, and citric acid monohydrate were ordered from Shanghai Guoyao Company (Shanghai, China). Cleanert C_18_ sorbent (40–60 µm, analytical grade) was purchased from Shanghai CNW Technologies (Shanghai, China). The centrifugal tubes, 1.5-mL vials, and 0.22-µm politetrafluoroetileno membranes were also obtained from Shanghai CNW Technologies (Shanghai, China).

### 4.2. Microorganism and Culture Medium

The *Edwardsiella ictaluri* strain was provided by Prof. Aihua Li from the Institute of Hydrobiology, Chinese Academy of Sciences (Wuhan, China). Brain–heart infusion broth used for culturing *E. ictaluri* was purchased from Qingdao Haibo Biotechnology Co. Ltd. (Qingdao, China).

### 4.3. In Vitro Susceptibility Testing

The minimum inhibitory concentration (MIC) was assayed in plasma using a micro-broth dilution method based on the Clinical and Laboratory Standards Institute (CLSI) recommended protocol. Briefly, serial 2-fold dilutions of DC from an initial concentration of 128 µg/mL were loaded into a 96-well microplate using plasma. Then the strain with a density of about 5×10^5^ CFU/mL in plasma was incubated with the drug for 24 h at 28 °C. The MIC was defined as the lowest concentration inhibiting bacterial growth.

### 4.4. Fish and Diet

Three hundred yellow catfish (120.2 ± 15.3 g, 48 months of age, male) were purchased from the culture facility of the Yangtze River Fisheries Research Institute (Wuhan, China). Every 18 fish were held in one tank (volume of each tank: 480 L) and acclimatized for 14 d at a water temperature of 24.0 ± 0.8 °C. The fish were fed with antibiotic-free feed that was made by the Nutritional Research Group in Yangtze River Fisheries Research Institute, Chinese Academy of Fishery Sciences, Wuhan, China. The feed contained 45.6% crude proteins, 6.3% crude fat, 8.4% moisture, 4.8% ash, and 0.4% total phosphorus [35]. The parameters of water quality were determined and maintained at the following status: Total ammonia nitrogen levels ≤ 0.74 mg/L, dissolved oxygen levels at 6.0–7.2 mg/L, pH at 7.1 ± 0.2, and nitrite nitrogen levels < 0.06 mg/L. The blank samples including blood, liver, kidney, muscle and skin, and gill were collected from 15 fish to establish the analytical method of ultra-performance liquid chromatography (UPLC) for DC before the formal experiment. All animal experimental protocols were approved by the Fish Ethics Committee of Yangtze River Fisheries Research Institute, Chinese Academy of Fishery Sciences, Wuhan, China.

### 4.5. Drug Administration and Sampling 

The detailed procedures are referred to our recent studies [36,37]. In brief, the fish were divided into three groups that were treated with three different single doses of DC at 10, 20, and 40 mg/kg, respectively, by oral gavage. Before giving the drug, DC powder was used to prepare the solution at a final concentration of 10 mg/mL. The DC solution was administered to each fish using a hard plastic tube attached to a 1-mL micro-injector. After oral gavage, if the fish regurgitated the given DC solution, the fish was removed from the tank and replaced by another. Blood samples were collected from the caudal vessels of each fish at the time points of 0.083, 0.17, 0.5, 1, 2, 4, 6, 8, 12, 16, 24, 48, 72, and 96 h after oral administration. After blood collection, each fish was dissected to collect liver, kidney, muscle and skin, and gill. Plasma samples were obtained by centrifugation of the corresponding blood samples at 1500 g for 5 min, and stored at −20 °C until analysis. The number of the plasma and tissue samples was n = 6 fish at each sampling time point.

### 4.6. Sample Preparation and Instrument Analysis

The method of sample preparation and the conditions of instrument analysis were also based on our previously reported procedures with some modifications [38]. In brief, 1 g of tissue samples (e.g., liver, kidney, gill, and muscle and skin) or 1 mL of plasma was thawed at room temperature and transferred into a 15-mL plastic tube. Then 5 mL McIlvaine buffer (0.04 mol/L sodium dihydrogen phosphate, 0.06 mol/L citric acid monohydrate, and 0.1 mol/L EDTA-Na_2_, pH = 4) was added to each tube and vigorously shaken for 30 s. After standing for 10 min, 4.5 mL of acetonitrile containing 3% formic acid was pipetted to each tube, and then 1 g of NaCl was weighted into them following shaking for 30 s. To ensure the maximum amount of the target compound was extracted from samples, the mixture of sample and extractant was sufficiently mixed by ultrasound for 5 min, and then centrifugated at 3500 g for 5 min. The resulting supernatant was decanted into a 10-mL tube. The above procedures were repeated. The obtained upper layer was combined into the same tube added 200 mg of C_18_ and 1 g of MgSO_4_ following shaking 30 s, and centrifuged at 3500 g for 5 min to remove impurities. The cleaned extractant was pipetted into a new 10-mL plastic tube and condensed to dryness by a gentle nitrogen stream at 45 °C. The dry extract was reconstituted by 1 mL 10% acetonitrile-water containing 0.1% formic acid. Finally, the mixture was filtrated by a 0.22 nylon member filter and prepared to analyze by UPLC. 

All samples were analyzed by a Waters UPLC (Milford, MA, USA) equipped with a binary solvent manager with a binary solvent pump, a sampler manager with an autosampler, and an ultraviolet detector. The detailed analytical conditions were set in line with our previous studies [15,16].

### 4.7. Calibration Curves and Recovery Rates

The collected blank samples of plasma, muscle and skin, liver, kidney, and gill were fortified with a standard solution of DC to get final concentrations of 50, 100, 500, 2000, 5000, and 20,000 μg/L or μg/kg. Samples were processed as described above, and each concentration was set with three parallels. Five replicates of spiked plasma and tissue samples at 50, 500, and 5000 μg DC /L or /kg were analyzed to calculate the precision and accuracy. 

### 4.8. Pharmacokinetic Analysis

The data of DC concentrations with time profiles in plasma and tissues were analyzed with a non-compartmental approach using Phoenix WinNonlin 8.0 (Certara, Inc., Princeton, NJ, USA). The prediction of PK profiles after multiple oral doses at different time intervals of dosage was performed using the non-parametric superposition approach [25]. The following PK parameters were calculated: λ_z_ (terminal rate constant), T_1/2λz_ (terminal half-life), T_max_ (time to observed maximal concentration after drug administration), C_max_ (observed maximal concentration), AUC_0–96_ (area under the concentration vs. time curve from 0 to 96 h), AUC_%extrap (percentage of AUC from time 0 to infinity due to extrapolation from the last observed time point to infinity), V_z__F (volume of distribution based on the terminal phase per fraction of dose absorbed), CL_F (total body clearance per fraction of dose absorbed), AUC_0–96_/MIC (the ratio of AUC_0–96_ to minimum inhibitory concentration), and %T > MIC (the percentage of the time profile of DC concentration more than minimum inhibitory concentration to total time).

## 5. Conclusions

This study determined the PK and PK/PD parameters of DC against *E. ictaluri* in yellow catfish based on MIC and PK studies after a single oral dose of 10, 20, and 40 mg/kg, respectively, along with a predictive methodology of a non-parametric superposition approach. The value of MIC was 500 µg/L in plasma. The increase of the oral dose enlarged the values of AUC_0–96_ in plasma and tissues except for liver, but other PK parameters had no apparent dose-dependence. The PK/PD parameter of AUC_0–96h_/MIC was prominently increased in plasma and each tissue (except liver) along with the rise of the DC dose, but the %T > MIC was only increased moderately at certain doses. Under the same oral dose with differing administration intervals of 96 to 12 h, the AUC_0–96h_/MIC was considerably increased in plasma and tissues, but the %T > MIC had a declined tendency. Finally, at 20 mg/kg with a time interval of 96 h, the %T > MIC reached from 99.5% to 100.0% in plasma and tissues, which could be the preferable dosage regime. Overall, this study provides some fundamental information on PK and PK/PD parameters to support the design of optimal therapeutic regimens of DC against *E. ictaluri* in yellow catfish.

## Figures and Tables

**Table 1:** Accuracy and precision of the method for doxycycline in fortified muscle and skin, liver, kidney, gill, and plasma samples of yellow catfish (*Pelteobagrus fulvidraco*).

Tissues or Plasma	Spiked Level (µg/kg or µg/L)	Recovery (%)	Within-Day RSD (%)	Between-Day RSD (%)
Muscle and Skin	50	77.3	4.4	5.1
	500	83.7	3.8	4.6
	5000	80.2	4.7	5.8
Liver	50	71.4	3.1	4.9
	500	72.7	4.0	6.2
	5000	67.2	4.3	5.8
Kidney	50	68.2	5.2	7.3
	500	71.7	2.7	4.5
	5000	79.7	2.2	3.7
Gill	50	80.3	3.5	5.5
	500	81.7	2.8	4.1
	5000	72.6	3.7	5.6
Plasma	50	82.7	4.5	5.7
	500	82.5	3.9	6.3
	5000	77.2	4.7	6.1

RSD: Relative standard deviation.

**Table 2:** The pharmacokinetic parameters of doxycycline in gill, kidney, liver, muscle and skin, and plasma of yellow catfish (*Pelteobagrus fulvidraco*) after a single oral dose of 10, 20, and 40 mg/kg, respectively.

Parameters	Unit	Gill			Kidney			Liver			Muscle and Skin			Plasma		
		10	20	40	10	20	40	10	20	40	10	20	40	10	20	40
λz	1/h	0.012	0.043	0.029	0.015	0.023	0.005	0.005	0.042	0.021	0.008	0.008	0.005	0.007	0.009	0.014
T1/2 λz	h	56.55	16.27	23.53	45.15	29.60	143.26	142.52	16.49	32.6	90.28	82.31	147.18	106.38	80.81	51.34
T_max_	h	0.50	0.50	0.50	1.00	0.50	2.00	1.00	24.00	1.00	6.00	4.00	12.00	6.00	4.00	12.00
C_max_	mg/kg (L)	0.66	120.74	151.94	1.03	11.64	15.40	1.08	34.81	24.95	0.18	2.30	2.84	0.44	4.67	6.99
AUC_0–96_	h*mg/L	44.69	708.96	1207.18	57.23	260.95	471.78	54.65	1615.75	790.04	15.22	86.52	126.48	27.86	192.10	223.56
AUC_%extrap	%	35.30	5.40	7.29	31.48	15.00	57.32	63.85	4.42	13.57	50.54	47.75	60.82	51.71	38.42	28.01
Vz_F	L/kg	NA	NA	NA	NA	NA	NA	NA	NA	NA	NA	NA	NA	26.60	7.47	9.54
Cl_F	L/h/kg	NA	NA	NA	NA	NA	NA	NA	NA	NA	NA	NA	NA	0.17	0.06	0.13

Notes: λz, the terminal rate constant; T1/2 λz, the terminal half-life; T_max_, the time to reach the peak concentration; C_max_, the peak concentration; AUC_0–96_, the area under concentration time curve from 0 to 96 h; AUC_%extrap, percentage of AUC from time 0 to infinity due to extrapolation from the last observed time point to infinity; Cl_F, the total body clearance per fraction of dose absorbed; Vz_F, the volume of distribution based on the terminal phase per fraction of dose absorbed; NA, not available or not applicable; *, a multiplication sign.

**Table 3:** The pharmacokinetic–pharmacodynamic parameters of doxycycline in gill, kidney, liver, muscle and skin, and plasma of yellow catfish (*Pelteobagrus fulvidraco*) given an oral dose of 10, 20, and 40 mg/kg at administration intervals of 96, 24, and 12 h, respectively.

Tissues and Plasma	Time Intervals (h)	Oral Doses (mg/kg)					
		10		20		40	
		AUC_0__–96_/MIC (h)	%T ˃ MIC (%)	AUC_0__–96_/MIC (h)	%T ˃ MIC (%)	AUC_0__–96_/MIC (h)	%T ˃ MIC (%)
Gill	96	89.37	43.99	1471.91	100.00	2414.36	100.00
	24	234.19	35.78	5037.98	96.00	7744.17	100.00
	12	425.79	21.94	9768.54	100.00	14677.36	100.00
Kidney	96	114.47	69.44	521.90	100.00	943.55	99.99
	24	304.91	65.56	1433.19	96.00	2707.67	99.99
	12	555.15	34.85	2660.07	94.84	4990.22	63.72
Liver	96	109.30	78.02	3231.50	99.98	1580.08	100.00
	24	284.60	71.70	9277.55	96.00	4548.55	100.00
	12	516.17	34.03	17116.13	23.59	8467.98	60.95
Muscle and Skin	96	30.43	NA	173.03	99.47	252.95	99.89
	24	79.25	NA	479.16	96.00	666.29	95.56
	12	143.61	NA	878.61	43.24	1213.31	40.54
Plasma	96	55.72	NA	384.21	99.90	447.12	99.94
	24	146.31	25.95	1087.35	96.00	1331.33	99.94
	12	265.29	12.99	1999.88	35.16	2461.86	53.31

Notes: AUC_0–96_/MIC, the ratio of AUC_0–96_ to minimum inhibitory concentration; %T ˃ MIC, the percentage of time profile of DC concentration more than minimum inhibitory concentration to total time; NA, not available or not applicable.

## Data Availability

All raw experimentally measured time versus concentration data of doxycycline in plasma, liver, kidney, gill, and muscle and skin of yellow catfish (*Pelteobagrus fulvidraco*) at different sampling times following a single oral dose of 10, 20, and 40 mg/kg are provided in Tables S1–S3, respectively, in the Supplementary Materials. All other calculated data are presented in the main text of this manuscript.

## Supplementary Materials

The following are available online at https://www.mdpi.com/2079-6382/10/3/329/s1, Table S1: The concentrations of doxycycline in plasma and tissues of yellow catfish (*Pelteobagrus fulvidraco*) after a single oral dose at 10 mg/kg at 24 °C; Table S2: The concentrations of doxycycline in plasma and tissues of yellow catfish (*Pelteobagrus fulvidraco*) after a single oral dose at 20 mg/kg at 24 °C; Table S3: The doxycycline concentrations in plasma and tissues of yellow catfish (*Pelteobagrus fulvidraco*) after a single oral dose at 40 mg/kg at 24 °C.
