# Supplementary material for: Determination of Pharmacokinetic and Pharmacokinetic-Pharmacodynamic Parameters of Doxycycline against Edwardsiella ictaluri in Yellow Catfish (Pelteobagrus fulvidraco)
Source: Antibiotics (Basel). 2021 Mar 21;10(3):329. doi: 10.3390/antibiotics10030329 (PMC8004065; doi:10.3390/antibiotics10030329)
Supplement: Supplementary file 1 [file antibiotics-10-00329-s001.pdf]

antibiotics

Supplementary Materials

Determination of pharmacokinetic and pharmacokinetic-pharmacodynamic parameters of doxycycline against *Edwardsiella ictalurid* in yellow catfish (*Pelteobagrus fulvidraco*)

Ning Xu [1,2,3], Miao Li [2], Xiaohui Ai [1,3,4,*], and Zhoumeng Lin [2,*]

1 Yangtze River Fisheries Research Institute, Chinese Academy of Fishery Sciences, Wuhan, 430223, China; xuning@yfi.ac.cn (N.X.); aixh@yfi.ac.cn (X.A.)

2 Institute of Computational Comparative Medicine (ICCM), Department of Anatomy and Physiology, College of Veterinary Medicine, Kansas State University, Manhattan, Kansas 66506, USA; xuning@yfi.ac.cn (N.X.); miaoli@ksu.edu (M.L.); zhoumeng@ksu.edu (Z.L.)

3 Hu Bei Province Engineering and Technology Research Center of Aquatic Product Quality and Safety, Wuhan, 430223, China; xuning@yfi.ac.cn (N.X.); aixh@yfi.ac.cn (X.A.)

4 Key Laboratory of Control of Quality and Safety for Aquatic Products, Ministry of Agriculture and Rural Affairs, Beijing, 100141, China; aixh@yfi.ac.cn (X.A.)

* Correspondence: zhoumeng@ksu.edu (Z.L.); aixh@yfi.ac.cn (X.A.)

Table S1, S2 and S3 showed the raw data of doxycycline concentrations in gill, kidney, liver, muscle+skin, and plasma of yellow catfish (*Pelteobagrus fulvidraco*) at different sampling times following a single oral dose of 10, 20, and 40 mg/kg, respectively.

Table S1. The concentrations of doxycycline in plasma and tissues of yellow catfish (*Pelteobagrus fulvidraco*) after a single oral dose at 10 mg/kg at 24 °C

Time (h)	Concentration (mg/kg or mg/L)				
	Gill	Kidney	Liver	Muscle+skin	Plasma
0.08	0.52±0.17	0.29±0.19	0.63±0.13	0.08±0.02	0.05±0.02
0.17	0.56±0.06	0.41±0.18	0.50±0.06	0.05±0.01	0.18±0.12
0.5	0.66±0.19	0.53±0.29	0.59±0.04	0.13±0.08	0.11±0.05
1	0.57±0.07	1.03±0.31	1.08±0.53	0.14±0.07	0.18±0.04
2	0.56±0.02	0.64±0.11	0.95±0.18	0.16±0.04	0.28±0.04
4	0.48±0.06	0.59±0.16	0.68±0.06	0.18±0.06	0.32±0.08
6	0.52±0.01	0.83±0.41	0.44±0.21	0.18±0.06	0.44±0.22
8	0.57±0.07	0.77±0.14	0.54±0.06	0.17±0.04	0.32±0.02
12	0.46±0.07	0.62±0.12	0.60±0.31	0.18±0.01	0.31±0.06
16	0.42±0.04	0.56±0.14	0.56±0.21	0.16±0.02	0.35±0.10
24	0.52±0.05	0.58±0.29	0.66±0.37	0.18±0.03	0.33±0.14
48	0.54±0.22	0.84±0.19	0.58±0.18	0.17±0.01	0.30±0.05
72	0.42±0.06	0.41±0.19	0.51±0.06	0.14±0.02	0.28±0.01
96	0.30±0.06	0.40±0.27	0.47±0.33	0.12±0.04	0.19±0.09

Table S2. The concentrations of doxycycline in plasma and tissues of yellow catfish (*Pelteobagrus fulvidraco*) after a single oral dose at 20 mg/kg at 24 °C

Time(h)	Concentration (mg/kg or mg/L)				
	Gill	Kidney	Liver	Muscle+skin	Plasma
0.08	18.19±12.66	10.54±7.75	2.01±0.90	0.08±0.03	0.47±0.10
0.17	81.82±20.88	8.21±3.38	5.20±1.38	0.21±0.12	0.71±0.59
0.5	120.74±20.31	11.64±0.96	8.78±2.45	0.50±0.13	0.94±0.37
1	56.37±19.98	7.61±4.46	10.02±3.12	0.57±0.21	2.12±0.26
2	94.89±39.66	5.87±0.69	23.52±5.55	0.88±0.73	2.44±0.49
4	45.78±21.71	5.67±1.88	24.15±15.43	2.30±0.83	4.67±2.97
6	24.53±14.54	9.82±6.62	16.32±1.01	1.08±0.47	1.99±0.61
8	17.20±5.67	4.34±3.05	10.16±2.07	1.46±0.58	2.81±1.10
12	15.11±3.83	1.96±1.47	19.96±11.21	0.93±0.57	3.78±1.40
16	9.53±4.24	2.16±0.78	15.49±6.36	1.03±0.27	2.41±0.14
24	2.87±1.96	1.84±0.42	34.81±10.22	1.39±0.84	3.35±1.96
48	2.56±1.52	3.32±2.61	23.62±9.87	0.86±0.55	1.55±0.14

72	0.73±0.34	2.18±0.68	5.10±3.20	0.52±0.46	1.32±0.40
96	1.73±0.15	1.08±0.33	3.14±1.79	0.67±0.28	1.03±0.12

Table S3. The doxycycline concentrations in plasma and tissues of yellow catfish (*Pelteobagrus fulvidraco*) after a single oral dose at 40 mg/kg at 24 °C

Time(h)	Concentration (mg/kg or mg/L)				
	Gill	Kidney	Liver	Muscle+skin	Plasma
0.08	20.87±8.22	4.47±2.90	15.21±9.16	1.38±0.48	0.77±0.41
0.17	94.59±19.27	3.22±0.72	13.97±9.84	0.33±0.12	0.98±0.63
0.5	151.94±76.89	8.85±1.66	14.93±9.29	1.26±0.63	1.54±0.64
1	67.83±34.52	10.5±5.95	24.95±8.73	1.24±0.24	2.87±1.41
2	105.94±68.08	15.4±9.79	24.14±7.44	1.19±0.86	5.04±2.83
4	14.26±3.24	6.88±4.45	11.17±5.38	1.31±0.25	3.62±1.49
6	38.7±20.85	7.38±2.37	24.07±10.34	2.09±0.59	5.89±2.72
8	23.62±12.46	12.12±8.43	16.91±7.56	1.85±0.66	4.45±3.16
12	50.40±15.04	9.19±3.59	23.38±4.76	2.84±1.28	6.99±2.30
16	20.35±11.34	7.97±2.16	12.57±7.12	1.43±0.56	3.75±1.18
24	6.12±1.93	4.49±1.83	9.92±4.09	1.57±0.88	2.82±1.16
48	8.58±3.77	3.74±2.06	2.49±0.71	0.87±0.46	1.42±0.82
72	3.12±1.61	3.68±0.77	7.68±2.54	1.40±0.81	1.53±0.41
96	2.80±0.84	3.07±0.39	2.64±1.07	0.92±0.14	1.17±0.64
